# Challenges and Opportunities for Strong and Dynamic Foundations in the Australian Cancer Plan

**DOI:** 10.5694/mja2.70331

**Published:** 2026-11-10

**Authors:** Ashley M. Hopkins, Andrew Rowland, Natansh D. Modi, Ceara Rickard, Mark Haseloff, Norma B. Bulamu, Imogen Ramsey, Niranjan Bidargaddi, Ganessan Kichenadasse, Michael J. Sorich, Kalinda E. Griffiths, Aideen McInerney‐Leo, David M. Roder, Raymond J. Chan

**Affiliations:** ^1^ Flinders Health and Medical Research Institute, College of Medicine and Public Health, Flinders University Adelaide South Australia Australia; ^2^ School of Pharmacy and Biomedical Sciences, Adelaide University Adelaide South Australia Australia; ^3^ Adelaide South Australia Australia; ^4^ Flinders Livewell Cancer Research Centre, Caring Futures Institute, College of Health and Enablement, Flinders University Adelaide South Australia Australia; ^5^ Flinders Medical Centre Adelaide South Australia Australia; ^6^ Dermatology Research Centre, Frazer Institute, University of Queensland Brisbane Queensland Australia; ^7^ Adelaide University Adelaide South Australia Australia

**Keywords:** artificial intelligence, benchmarking, cancer, clinical trials as topic, data collection, proteomics

## Abstract

Strategic Objective 4, ‘Strong and Dynamic Foundations’, is a structural enabler of the Australian Cancer Plan's decadal vision. Recent nationally endorsed frameworks and funded initiatives have signalled clear momentum in data standardisation, person‐centred measurement and precision oncology. This perspective article examines the structural barriers that continue to impede progress across connected data, precision omics, clinical trials, digital health, artificial intelligence and patient‐reported measures in Australian cancer care. Legislative and governance reform are critical bottlenecks to national data integration; genomic profiling inequities constrain equitable trial access; and digital health technologies remain trapped in perpetual pilot mode. Addressing these barriers through coordinated governance reform, sustained investment, workforce development and equity as a measurable default is essential to delivering improved cancer outcomes for all Australians.

## 1 Introduction

This perspective article is part of the *Medical Journal of Australia* series examining progress in implementing each Strategic Objective of the Australian Cancer Plan (ACP), along with the associated challenges and opportunities. Launched in November 2023, the ACP is a 10‐year national framework designed to improve cancer outcomes and experiences for all Australians affected by cancer, as well as their families and carers [1]. The ACP is structured around six Strategic Objectives that identify where national focus and effort are required: (i) Maximising Cancer Prevention and Early Detection; (ii) Enhanced Consumer Experience; (iii) World Class Health Systems for Optimal Care; (iv) Strong and Dynamic Foundations; (v) Workforce to Transform the Delivery of Cancer Care; and (vi) Achieving Equity in Cancer Outcomes for Aboriginal and Torres Strait Islander People [1]. This article focuses on Strategic Objective 4: Strong and Dynamic Foundations.

Strategic Objective 4, ‘Strong and Dynamic Foundations’, is an enabler of the ACP [1, 2]. It sets out a 10‐year ambition for a modern, fit‐for‐purpose cancer control infrastructure using technology, research and connected data. Realising this ambition will require implementation of a nationally agreed framework for comprehensive data collection and reporting, integration of new technologies into routine care and an innovation‐ready ecosystem that can measure and deliver equitable care, including access to clinical trials.

This perspective article examines the structural barriers that continue to impede national infrastructure reform and proposes practical solutions across key foundational pillars: connected data, precision omics, novel therapeutics, digital health, artificial intelligence (AI) and patient‐reported outcome measures (PROMs) and patient‐reported experience measures (PREMs) [1, 2, 3]. The aim is to make equity the default, measurable outcome of Australian cancer care.

## 2 Progress in Connected Data, Person‐Centred Measurement and Precision Oncology

Over the past 2 years, broad ambition has begun to translate into nationally endorsed frameworks and funded initiatives. A key milestone is the National Cancer Data Framework (NCDF), which sets the strategic direction and priorities for collecting, managing and using cancer data across Australia [4]. The NCDF aims to support a mature cancer data ecosystem that enables performance reporting and benchmarking, embeds Indigenous data sovereignty, underpins data‐driven improvements in patient care and supports world class research [4, 5].

The Australian Government has also announced a $1.5 million investment in the Australian Institute of Health and Welfare to establish the Australian Cancer Data Alliance and strengthen jurisdictional cancer registries, with the aim of accelerating the national collection of cancer stage and recurrence data [6]. In parallel, a systemic shift towards patient‐centred measurement is underway through the Australian Real World Cancer Evidence Network (Pan Cancer Initiative) [7, 8]. This $22.5 million co‐investment partnership between Movember and Cancer Australia is promoting routine collection and use of PROMs and PREMs through a national electronic platform that supports secure, scalable capture in settings such as clinical quality registries [7, 8]. The Pan Cancer Initiative is a key operational mechanism for delivering an enhanced consumer experience, but only if PROMs and PREMs are available in real time and linked to processes that enable clinical response.

Clinical trial infrastructure is also being strengthened through the continued Support for Cancer Clinical Trials program (2024–2027), which has awarded $22 million to the 14 Multi‐site Collaborative Cancer Clinical Trials Groups, and through the $3 million Cancer Genomics Clinical Trials Fund to accelerate incorporation of genomics into trials, research and care [9, 10].

These initiatives signal momentum in data standardisation, digitally enabled person‐centred care and precision oncology. The critical next steps are to translate frameworks into measurable change in routine practice, operationalise across a federated system, ensure Aboriginal and Torres Strait Islander peoples' data sovereignty and achieve demonstrable equity in access to quality care and clinical trials.

## 3 Challenges and Opportunities

Australia now has a clearer national direction, but direction alone will not deliver system change. The immediate challenge is to convert endorsed frameworks into routine infrastructure that is usable across jurisdictions, visible in practice and accountable for equity. Tables 1 and 2, along with the accompanying narrative, provide complementary perspectives on the investment, research and implementation priorities needed to translate this direction into practice.

**TABLE 1:** Investment priority areas to accelerate early progress towards Strategic Objective 4 of the Australian Cancer Plan.

System gap	Investment priority area	Stakeholder perspectives	Forward‐looking statement
Governance and ethics constrain data linkage.	Reform to establish common rules for federated linkage, access, ethics and governance.	Trusted sharing and accountability arrangements are required.	Create the foundation for routine national data linkage and safe technology scale‐up.
Cancer data are fragmented across systems.	National Cancer Data with agreed core variables, definitions and federated pipelines.	Core data are required for benchmarking, care monitoring and trial matching.	Build a usable national data asset for real‐world evidence and value‐based assessment.
Poorer outcomes and inequitable care for Aboriginal and Torres Strait Islander peoples are not consistently measurable.	Enable Aboriginal and Torres Strait Islander peoples‐led cancer data governance and equity reporting.	Communities need control and culturally safe data use and reporting.	Make equity measurable and strengthen trust and accountability.
PROMs and PREMs are not routinely linked to real‐time response or benchmarking.	National PROM/PREM infrastructure.	Patients want symptoms and experiences acted on; services need workable digital workflows.	Embed person‐centred measurement in routine cancer care nationally.
Access to genomic profiling and clinical trials remains uneven.	Equitable genomic profiling, trial matching and teletrial access.	Patients need timely genomic testing and trial opportunities regardless of postcode.	Make precision oncology, trial matching and teletrial participation accessible to all Australians.
Insufficient support can limit the integration of data, genomics, trial and AI/digital infrastructure.	Workforce, digital capability and implementation support.	Services need funded roles and training to operationalise new tools, not just access to them.	Build the workforce and capability to embed effective infrastructure in routine care.
Infrastructure sometimes remains in pilot mode or is withdrawn before its value is realised.	Sustainability planning and long‐term infrastructure stewardship.	Confidence that infrastructure delivering value will be sustained.	Build stewardship and funding pathways that sustain infrastructure.

Abbreviations: AI, artificial intelligence; PREM, patient‐reported experience measure; PROM, patient‐reported outcome measure.

**TABLE 2:** Research priority areas to accelerate early progress towards Strategic Objective 4 of the Australian Cancer Plan.

Evidence gap	Research priority area	Stakeholder perspectives	Forward‐looking statement
Effective strategies for cross‐jurisdictional cancer data linkage are not yet set.	Governance, ethics and custodial models for federated cancer data linkage and sharing.	Governments and custodians need secure, practical and acceptable processes.	Deliver a blueprint for cross‐jurisdictional cancer data linkage, access and reuse.
Processes for core variable, completeness, timeliness, linkage quality and fitness for care need validation.	Validate technological processes for dataset formatting and access.	Datasets need to be complete, accurate and timely enough for care and system improvement.	Define measurable standards for national cancer data quality and utility.
Best approach to operationalise Aboriginal and Torres Strait Islander peoples‐led data sovereignty for cancer data.	Aboriginal and Torres Strait Islander peoples‐led data standards and processes that make equity of care measurable and actionable.	Must be community‐led and deliver usable data for communities, services and benchmarking.	Establish community‐led processes that support benchmarking and drive measurable improvements in outcomes.
The best approach to PROM/PREM capture, response, benchmarking and health economic use remains uncertain.	National PROM/PREM capture, response and health economic measurement models.	Stakeholders need evidence that routine measurement is linked to action, improves care and is sustainable.	PROMs and PREMs embedded in care that support benchmarking and improve patient outcomes.
Optimal models for timely genomic testing, real‐time data use, automated trial matching and teletrial delivery remain uncertain.	Evaluate implementation strategies for equitable precision omics, trial matching and teletrial delivery.	Patients, clinicians and trial groups need cost‐effective models that improve equitable access to trials and precision oncology.	Effective and scalable delivery of precision oncology and real‐time trial offers that drive care improvements for all.
Optimal ways to fund specialist roles and upskill the workforce for data, genomics, trial and AI/digital care remain unclear.	Evaluate workforce and training models for routine use of data, genomics, trial access and AI/digital care.	Services need evidence on which roles, training and supports improve uptake, equity and workload.	Define best‐practice workforce and training models for routine data, genomics, trial access and AI/digital care.
Evidence on sustainable stewardship models for cancer infrastructure is limited.	Evaluate sustainable funding, stewardship and value‐demonstration models for data, genomics, trials and digital infrastructure.	Governments, patients, communities and payers need safe, equitable and sustainable models.	Define funding and stewardship models that sustain infrastructure, demonstrate value and guide reinvestment.

Abbreviations: AI, artificial intelligence; PREM, patient‐reported experience measure; PROM, patient‐reported outcome measure.

### 3.1 Policy, Governance and a Minimum Dataset for Learning, Accountability and Improvement for All

Despite the NCDF, Australia's cancer data remain fragmented across jurisdictions, public and private custodians and siloed sources such as screening registers, pathology services, hospital systems and clinical registries. Gaps and inconsistencies continue to hinder a comprehensive, longitudinal national view of cancer control, patient pathways and outcomes. Further, although population‐based cancer registries provide a strong foundation for incidence and mortality reporting, nationally consistent data on patient characteristics, stage at diagnosis, treatment, recurrence, hospital utilisation and patient‐reported measures are not yet routinely available at scale. This limits benchmarking, assessment of adherence to optimal care pathways and quantification of variation and inequity in cancer care and outcomes.

These limitations are particularly pronounced for Aboriginal and Torres Strait Islander peoples, for whom incomplete and inconsistent identification within cancer datasets constrains visibility in reporting and limits the capacity to monitor outcomes and service delivery [11]. Cancer data systems must embed Indigenous data governance aligned with national priorities, including the National Agreement on Closing the Gap and the Framework for Governance of Indigenous Data, enabling Aboriginal and Torres Strait Islander leadership in decisions regarding community‐prioritised measures, data collection, linkage, interpretation and use [12, 13, 14].

Notably, high‐quality registry and linked datasets are essential for service planning, monitoring the translation of evidence‐based interventions into routine practice, evaluating service performance and conducting descriptive observational research at scale. They enable a learning and accountable health system to understand real‐world treatment patterns, resource use, uptake of evidence‐based interventions, variation in care and long‐term outcomes beyond clinical trials and to demonstrate value across new therapeutics, service delivery models, genomics integration, digital health interventions and trial infrastructure. They should be viewed as central to the sustainability of care. Without robust, routinely analysed registry and linked data, the system cannot quantify return on investment, identify where implementation is incomplete or inequitable, support continuous quality improvement or justify ongoing programmatic funding such as the $264 million Lung Cancer Screening Program and the $166 million Cancer Nursing and Navigation Program [15, 16].

Therefore, the immediate priority is to implement the NCDF by providing timely, connected and standardised data for clinicians, health services, researchers, quality‐improvement programs and national benchmarking. Such actions must be supported by streamlined policies, governance and ethics processes. At the core of these policies is the need to mandate a minimum targeted cancer dataset with agreed core variables, definitions and governance arrangements. These datasets should include standardised demographics and equity population characteristics (including identifiers), stage at diagnosis, key treatment milestones, hospital admissions and activity, recurrence or progression where feasible, survival outcomes and patient‐reported measures (including measures suitable for health economic analysis), along with comorbidities and biobanking data where available. The case for such a nationally linked, de‐identified longitudinal data asset has long been made across the cancer sector; however, realising this vision depends on coordinated implementation across jurisdictions, with sufficient policy levers and resourcing commitments in place. Australia's experience with the Australian Immunisation Register provides a narrower but useful precedent: mandated reporting and regular public coverage reporting can make population‐level performance visible, strengthening accountability and the case for sustained investment [17].

Technical capability, including federated approaches that enable analysis across distributed datasets, is unlikely to be the main barrier to data integration. Rather, a minimum‐targeted cancer dataset should be designed so that core variables are captured through routine clinical workflows, drawn where possible from existing clinical systems and linked to administrative data from the Medicare Benefits Schedule (MBS) and Pharmaceutical Benefits Scheme (PBS), without duplicative manual entry. The larger constraints are legislative, governance and custodial arrangements across a segmented health system, where multiple custodians and differing jurisdictional requirements impede timely cross‐border linkage, access and reuse. Commonwealth, state and territory health departments must therefore commit, with defined timelines, to cancer data sharing and linkage as the default in a federated model, supported by consistent standards, security controls, clear access pathways, legislative support and service‐level agreements that enable governed use for quality assurance, service improvement, policy and research.

With secure, near real time data flows and Aboriginal and Torres Strait Islander peoples‐led data governance, a minimum targeted dataset could become a practical foundation for a National Cancer Dataset that supports system learning, targeted improvement, accountability, person‐centred care and more efficient and equitable access to clinical trials and benchmarking of care. International experience shows what becomes possible when data linkage, governance and access pathways are treated as core system functions: Denmark and other Nordic countries have long demonstrated the value of population registries, personal identifiers and structured access arrangements [18, 19, 20], whereas Scotland's Safe Haven Network and Health Data Research Network Canada show how secure, governed access and linkage can be coordinated across distributed regional, provincial and national data custodians [21, 22].

### 3.2 Equitable Access to Clinical Trial and Precision Omics

Clinical trials underpin oncology innovation and can provide access to emerging therapeutics and interventions [23]. Yet substantial geographical, socio‐economic and institutional inequities persist across the Australian trial landscape [24, 25]. These gaps matter not only because they restrict participation, but because they shape the evidence base for routine care [24, 25]. When priority populations are underrepresented, trial findings may be less applicable across Australian patients and care settings, and innovation may be developed, validated and implemented in ways that do not reduce and may inadvertently exacerbate, existing inequities.

The structural drivers are well recognised. Trial infrastructure and specialised research workforces remain concentrated in metropolitan tertiary hospitals, limiting participation for rural and remote communities. Language and cultural barriers for culturally and linguistically diverse communities and Aboriginal and Torres Strait Islander peoples continue to limit equitable access and impede the provision of informed and culturally safe consent. Although trial‐level barriers include eligibility criteria and protocol requirements that may limit participation for people with comorbidities, inadequate organ function, limited English proficiency or other support needs [25]. These barriers are further obscured by the absence of routinely collected and consistently reported demographic and population identifiers within clinical trial registries and datasets, limiting the ability to monitor who is offered, enrolled in and retained in trials and where inequities persist [26, 27].

Precision oncology is a related but distinct foundation of equitable cancer care, increasingly reliant on comprehensive genomic profiling to guide targeted therapy and determine eligibility for many contemporary trials. Where the benefit of omics‐guided approaches over standard care remains uncertain, prospective evaluation, including randomised or other comparative designs where feasible, is needed to support evidence‐based implementation. However, access to advanced genomic testing remains uneven and is not consistently embedded as a routine, affordable component of cancer care [28, 29]. This represents a diagnostic bottleneck: genomics cannot support equitable trial access if the sequencing needed for identification and matching is inaccessible to priority populations [30]. Nonetheless, the Omico PrOSPeCT program [31] and its Cancer Screening Program (CaSP) [32] demonstrate that a nationally decentralised model for comprehensive genomic profiling is being built, despite their scope currently being limited to advanced and incurable cancers. However, for Aboriginal and Torres Strait Islander peoples, other challenges to equitable precision oncology eclipse access. Specifically, the ability to interpret and identify therapeutic targets for germline and somatic genetic variants is limited by a lack of reference databases for Aboriginal and Torres Strait Islander peoples. Emerging data show that some germline alterations in Aboriginal and Torres Strait Islander peoples have not been previously described in genomic databases, and thus, the clinical significance is unknown [33]. These data gaps reinforce the importance of Aboriginal and Torres Strait Islander peoples‐led genomic data governance initiatives such as the National Centre for Indigenous Genomics (NCIG) and the Australian Alliance for Indigenous Genomics (ALIGN) [33, 34].

An equity‐driven, federated national cancer dataset that is accessible in near real time is important not only for benchmarking and system learning but also for timely trial–patient matching at the point of care. Such matching requires high‐quality clinical and genomic data within real‐world data systems, supported by automated trial‐matching technologies that draw on trial‐offering registries and provide clinician and patient decision support [35]. Alongside this, sustained investment is required in networked and decentralised trial delivery models, including teletrial hub‐and‐spoke approaches [36].

Overall equity must be measured. Health networks should embed routine trial offering into standard care and mandate transparent, facility‐level reporting of trial offerings and enrolment rates, stratified by remoteness and other priority group characteristics. They should also monitor whether the genomic testing needed for trial matching is being offered, completed and returned in time to support care for all relevant patients. Equitable trial access cannot be achieved if access to the underlying genomic information remains uneven.

## 4 System Enablers for Sustainable Implementation

These priorities will not be implemented at scale without enabling conditions within the health system itself. Workforce capability, funding reform, integration beyond tertiary care and a shift from pilots to permanent, sustainable digital infrastructure are therefore additionally critical.

### 4.1 Workforce Capability, Data Literacy and Implementation Support

Infrastructure reform must be matched by deliberate investment in workforce capability. High‐quality cancer data, genomics and AI systems will have limited impact without clinicians, data scientists, trial coordinators and informatics specialists embedded within health services to operationalise them. Australia requires a coordinated strategy to build cancer data literacy across the workforce, expand genomics‐informed clinical capacity within and beyond metropolitan tertiary centres and develop sustainable career pathways in clinical informatics and cancer data science. Regional services, in particular, require funded trial coordinators and human and digital navigation support to actively reduce inequity. As AI use expands in healthcare, improving AI literacy among cancer care providers will be essential to realising capacity gains.

### 4.2 Funding Reform, Incentive Alignment and Sustainability

Sustainability will depend on aligning funding models with infrastructure ambitions. Routine patient‐reported measure collection, genomic interpretation, trial matching and data reporting require time, workforce and digital infrastructure that are not consistently recognised or reimbursed under current activity‐based funding and MBS/PBS arrangements. These ambitions also require follow‐on care. For example, patient‐reported measures must be followed by self‐management support and clinical actions where required. If these activities remain unfunded or peripheral, they risk remaining aspirational rather than embedded. Commonwealth and state funding frameworks must explicitly support data capture, genomic review processes, decentralised trial delivery, quality reporting and data sharing as core components of cancer care. Infrastructure reform without reimbursement reform will struggle to achieve durable system‐wide implementation.

ACP Action 4.5.3 explicitly calls on the system to ‘explore and test innovative approaches to health service funding models to address areas of need, and system improvement, in cancer care’ [1], recognising that equitable, priority‐driven and sustainable funding is critical for high‐performing cancer care regardless of cancer type, geographical location or population group. The next step is to translate this principle into concrete mechanisms that sustainably embed these activities in routine care.

### 4.3 Integration With Primary Care Across the Cancer Continuum

A strong national cancer data foundation must extend beyond acute tertiary treatment settings to encompass primary care, early diagnosis, survivorship and long‐term outcomes. Linked datasets should capture the full cancer pathway, including symptom presentation and referral in general practice, treatment coordination, late effects, comorbidities, psychosocial outcomes, return‐to‐work trajectories and ongoing care between oncology and general practice. Without integration of general practice and community‐based data, Australia risks constructing an infrastructure that measures treatment episodes rather than whole‐of‐life outcomes. Denmark and other Nordic countries, including Finland, Sweden and Norway, illustrate this well: individual‐level linkage across general practice records, national registries and socio‐economic data has enabled researchers to measure the population‐level impact of the full cancer pathway from primary care presentation and referral through to survival and long‐term outcomes [18, 19, 20]. A longitudinal, cross‐sector dataset is essential to ensure equity and quality are sustained across the cancer control continuum.

### 4.4 Escaping ‘Pilot Mode’ in Digital Health and AI Integration

The ACP explicitly highlights digitally enabled care, including virtual navigation, remote patient monitoring and AI, as a vital mechanism for improving equity and access, particularly for geographically dispersed regional, rural and remote communities [2]. These technologies are also critical to realising the NCDF. However, although some digital health innovations have been scaled nationally, many remain difficult to translate from controlled experimental settings into sustained, system‐wide infrastructure. Without sustained programmatic funding and cohesive governance, effective interventions risk remaining trapped in perpetual ‘pilotitis’, unable to progress to system‐wide adoption [37].

The critical opportunity over the next decade is to invest in adaptable, standards‐based digital infrastructure across a cohesive national cancer network. Transitioning from isolated pilot projects to permanent clinical infrastructure will require replacing short‐term discovery grants with sustainable, programmatic funding models embedded within core health system operating budgets. Without these investments, underresourced regional services risk falling further behind. To avoid fragmented local adoption of preferred tools, implementation should be clinically led and coordinated across services, with sector agreement on minimum standards for tool selection, interoperability, evaluation and monitoring, while allowing appropriate local adaptation. Governance frameworks must also be rigorously developed and enforced to enable safe, system‐level implementation of AI and virtual navigation tools that have demonstrated cost‐effectiveness across a national network. Such governance mechanisms must include clinical leaders, cancer care providers, those affected by cancer (patients and carers) and data and AI experts.

## 5 Conclusion

Realisation of the ACP depends on achieving Strategic Objective 4, ‘Strong and Dynamic Foundations’, because it is a structural enabler of the Plan's decadal vision of world class cancer outcomes and experiences for all Australians. In Tables 1 and 2, we present a scenario for how focused investment, directed across investment and research priorities, could accelerate early progress towards this objective, recognising it as an initial stimulus for sustained commitment. Realising the full 10‐year ambition will require Australia to implement the NCDF in a way that delivers near real time, longitudinally linked clinical data, governed through robust, well‐funded, Aboriginal and Torres Strait Islander peoples‐led data sovereignty arrangements. Clinical trials and precision genomics must be integrated into routine clinical care through systems that enable equitable, decentralised trial matching, thereby reducing the postcode lottery of cancer survival. The health system must also break the cycle of digital health and AI pilots by committing to sustainable programmatic funding and scalable governance. Through targeted investments in connected data, precision omics, digital health and AI, and patient‐reported measures, supported by systematic use of registry and administrative datasets for planning, monitoring and real‐world observational research, Australia can build a modern, fit‐for‐purpose cancer care ecosystem that delivers measurable outcomes for all Australians and sustains value over time.

## Author Contributions

Ashley M. Hopkins, Andrew Rowland, Natansh D. Modi, Ceara Rickard, Mark Haseloff, Norma B. Bulamu, Imogen Ramsey, Niranjan Bidargaddi, Ganessan Kichenadasse, Michael J. Sorich, Kalinda E. Griffiths, Aideen McInerney‐Leo, David M. Roder and Raymond J. Chan contributed to conceptualisation, investigation, writing – original draft and writing – review and editing.

## Funding

Ashley M. Hopkins holds an Emerging Leader Investigator Fellowship from the Australian National Health and Medical Research Council (APP2008119), along with other active funding from Flinders Foundation and Tour de Cure, Australia.

Norma B. Bulamu holds an Early Career Researcher Fellowship from Cancer Council South Australia (formerly Cancer Council SA Beat Cancer Project). The funders had no role in the conception, design, analysis, interpretation or writing of the manuscript and had no influence over the content or conclusions.

## Disclosure

Not commissioned; externally peer reviewed.

## Conflicts of Interest

Ashley M. Hopkins is a recipient of investigator‐initiated funding for research outside the scope of the current study from Boehringer Ingelheim; and is a recipient of speaker fees from AstraZeneca. Andrew Rowland is a recipient of investigator‐initiated funding for research outside the scope of the current study from AstraZeneca, Boehringer Ingelheim, Pfizer and Takeda; a recipient of funding for research outside the scope of the current study from the Australian National Health and Medical Research Council; and is a recipient of speaker fees from Boehringer Ingelheim and Genentech. Raymond J. Chan is a Chair of Research and Data Advisory Committee and is a member of the 2‐year Evaluation of the Australian Cancer Plan Expert Advisory Group of Cancer Australia. Cancer Australia was given the opportunity to review this manuscript for factual accuracy. Cancer Australia had no role in the conception, design, analysis, interpretation or writing of the manuscript and had no influence over the content or conclusions. The remaining authors have no other potential conflicts of interest to disclose.

## Data Availability

This article includes no original data.
